# Sex Differences in White Matter Diffusivity in Children with Developmental Dyslexia

**DOI:** 10.3390/children11060721

**Published:** 2024-06-13

**Authors:** Gehna Gupta, C. Nikki Arrington, Robin Morris

**Affiliations:** 1 Department of Neuroscience, Georgia State University, Atlanta, GA 30303, USA; ggupta1@gsu.edu; 2 Georgia State/Georgia Tech Center for Advanced Brain Imaging, Atlanta, GA 30318, USA; robinmorris@gsu.edu; 3 Department of Psychology, Georgia State University, Atlanta, GA 30303, USA; 4 Center for Translational Research in Neuroimaging and Data Science, Atlanta, GA 30303, USA

**Keywords:** developmental dyslexia, reading, white matter, sex differences, reading network, DTI, reading disability, neurobiology, pediatrics, learning disability

## Abstract

Despite the high prevalence of developmental dyslexia in the U.S. population, research remains limited and possibly biased due to the overrepresentation of males in most dyslexic samples. Studying biological sex differences in the context of developmental dyslexia can help provide a more complete understanding of the neurological markers that underly this disorder. The current study aimed to explore sex differences in white matter diffusivity in typical and dyslexic samples in third and fourth graders. Participants were asked to complete behavioral/cognitive assessments at baseline followed by MRI scanning and diffusion-weighted imaging sequences. A series of ANOVAs were conducted for comparing group membership (developmental dyslexia or typically developing), gender status (F/M), and white matter diffusivity in the tracts of interest. The Results indicated significant differences in fractional anisotropy in the left hemisphere components of the inferior and superior (parietal and temporal) longitudinal fasciculi. While males with dyslexia had lower fractional anisotropy in these tracts compared to control males, no such differences were found in females. The results of the current study may suggest that females may use a more bilateral/alternative reading network.

## 1. Introduction

Developmental dyslexia (DD) is a neurodevelopmental disorder characterized by deficits in phonological and orthographic processing of written words that affects approximately 5–15% of the U.S. population [1]. Phonological processing refers to the decoding of words or sentences by speech sounds, while orthographic processing is the visual processing of the letter regularities within a word. When learning how to read, children are required to match the sounds of syllables (phenomes) and the printed letters of a word (visual print) with its corresponding meaning (semantics). Because children with DD have trouble achieving this integrative task, they show atypical and unexplained difficulties in reading fluency and accuracy, often affecting their academic achievement and self-esteem [2].

### 1.1. The Reading Network: The Healthy Brain

The understanding of symptoms indicative of DD requires the study of the process of reading, a learned skill that utilizes several visual, linguistic, cognitive, and attentional processes [2]. Researchers have found that unlike other biological processes, reading is an acquired skill requiring active learning and guided teaching [3]. Children start by becoming aware of the phonological sounds that accompany words and then mapping them onto the written word. This phonological–orthographic mapping is one of the key components of efficient reading and instruction. Neuroimaging research has been able to identify key areas in the brain that help connect these phonemic, orthographic, and semantic lexicons (mental representation of words) together, including the frontal, tempo-parietal, and the occipito-temporal regions in the left hemisphere of the brain [4]. The brain communicates and processes information through the complimentary functioning of gray matter, neuron cell bodies where the processing of information takes place, and white matter, myelinated axon bundles that connect neurons in different brain regions into functional units [5,6]. Efficient reading skills require adequate development and utilization of not only brain areas that are important for reading, but also the white matter tracts that subserve the reading network, working to communicate effectively between the brain regions and networks that specialize in language processing and reading. White matter tracts along with the gray matter cortical regions in the left hemisphere together form a left hemispheric network, also known as the reading network, that consists of a dorsal and a ventral route [4].

This dual-route system is believed to be at work in many of the brain’s cognitive processing tasks, such as the dorsal “where” and ventral “what” streams in the visual pathway. Functional MRI studies have highlighted the different functions of dorsal and ventral reading streams. The dorsal reading route is located in the tempo-parietal region of the brain and is constituted by the left posterior superior temporal gyrus (lpSTG), inferior parietal lobe (IPL), and other inferior frontal and temporoparietal regions, along with the superior longitudinal fasciculus (SLF) that connects these areas [4,7]. In addition, research has found that brain connectivity in the left temporo-parietal region correlates with reading abilities in children as young as 8 to 12 years [8], highlighting the significant role of this left lateralized dorsal reading network in the development of efficient reading skills. Research has outlined the dorsal reading network’s primary involvement in phonological processing, along with the integration of visual and auditory information [9]. Researchers have gone as far as to propose that reading acquisition starts with the emergence of this dorsal reading circuit, which then aids in skills necessary for phonological processing [9,10,11]. The strength of the connections between the left inferior parietal cortex and the left posterior occipito-temporal cortex in pre-readers has been shown to predict reading skills at a later stage, emphasizing the dorsal phonological reading network’s critical role in reading acquisition [11].

As children learn to read, they show a gradual shift from this dorsal route of sound–symbol and phonological decoding towards more automatic word recognition using the ventral route. The ventral route is proposed to be important for the fast and automatic processing of whole words that is necessary for fluent reading [9]. The ventral reading route runs along the occipito-temporal circuit, encompassing the fusiform gyrus, the inferior occipital gyrus, and white matter tracts such as the inferior longitudinal fasciculus (ILF), inferior frontal occipital fasciculus (IFOF), and uncinate fasciculus (UF) [4,11]. This ventral network also encompasses the Visual Word Form Area (VWFA), a part of the brain that becomes specialized in the visual processing of words and their common letter patterns within a specific language [12]. Some researchers have found that the neurons in the VWFA have a preferential response to real words compared to other letter strings [11,13]. The ventral reading network, including the VWFA, is thus proposed to function as a highly specialized network for fast and automated reading of written words.

While there is a limited number of longitudinal brain imaging studies that examine this dorsal-to-ventral shift hypothesis directly, some studies do show a greater reliance on the ventral reading stream as children become more experienced readers [11,14,15,16]. At the same time, this hypothesis should not be taken to mean that only one reading stream is active in the brain at a time. Instead, researchers propose that both the dorsal and the ventral reading streams are utilized in an integrated way by the brain depending on the type of words being read, along with the reading skills, age, and experience of the reader [11].

#### 1.1.1. White Matter Tracts Supporting the Reading Network

As previously reported, there are key white matter tracts that subserve the dorsal and ventral brain areas of the reading network: the SLF, which functions as the key dorsal white matter tract; the ILF and UF that subserve the ventral route of the reading network; and the CC that connects the brain’s two hemispheres. These key white matter tracts are critical in the development of the efficient communication between various brain regions required for efficient reading.

##### Superior Longitudinal Fasciculus

The SLF connects the tempo-parietal language regions to the ipsilateral frontal regions [17]. There is not a clear scientific consensus on the differentiation of the SLF, and parts of the SLF are often used interchangeably with the direct and indirect bundles of the arcuate fasciculus in the neuroscientific literature [18]. Based on DWI, other authors and researchers advocate for the existence of two main SLF bundles: anterior/dorsal and posterior/ventral [19]. The fact that the SLF comprises different segments implies that each segment might underlie distinct functions [17].

In more reading and language processing-related research, SLF IV, corresponding to the dorsal portion of the SLF (connecting the frontal and ocular areas to the superior temporal gyrus), has been given specific attention, given its connections between important areas of language production and comprehension: Broca’s area and Wernicke’s area [17]. The SLF IV component, also commonly referred to as the arcuate fasciculus (AF), is believed to be a component of the key phonological processing pathway [4]. This is supported by fMRI studies that imply word reading to be a grapho-phonological process involving both the tempo-parietal junction and inferior frontal regions connected by the AF [4,20]. This view is also supported by lesion studies showing that damage to the AF/SLF IV is associated with impairment of phonological skills and acquired reading impairment [21].

##### Inferior Longitudinal Fasciculus

The ILF connects the occipital cortex to the anterior temporal lobe and subserves the ventral reading network. More precisely, it runs from the extra striate visual association areas and connects the fusiform gyrus and the cuneus with further coupling of the lateral and medial anterior temporal regions [4]. While the function of the ILF has been debated and lacks a clear consensus, studies have suggested that it is important in connecting the occipital areas of the brain to the VWFA and is thus proposed to have important functions regarding visual memory and perception of word patterns [4,22,23]. The ILF’s connection to the VWFA and the occipital cortex, along with its function in visual word perception, gives reasonable cause to place it as a key part of the brain’s orthographic word processing route.

##### Uncinate Fasciculus

The UF connects the orbitofrontal cortex to the anterior temporal lobe [24]. There exist conflicting reports on the importance of UF for reading and language processing. Previously, the UF was believed to be essential for language processing since it connected language-supporting areas in the frontal and temporal cortex, thus considered essential for encoding, storing, and retrieving word and semantic knowledge [25,26,27]. However, some studies on the anatomy of the UF show that it may not link any specific areas that are important in language processing, but instead connects brain regions in the ventral and medial frontal lobe to the anterior temporal lobe and surrounding areas [27]. Despite this, research has shown a correlation between a higher fractional anisotropy in the UF with reading comprehension and verbal memory, suggesting the tract’s function in processing complex or higher-level elements of reading [28,29,30]. While the conflicting literature makes the role of UF in language and reading processes somewhat ambiguous, the tract’s anatomical position in connecting the temporal and orbitofrontal lobe along with its role in reading comprehension make it a reasonable subject of ongoing research.

##### Corpus Callosum

As the largest white matter bundle in the brain, the CC works to connect the two hemispheres and can be divided into anterior, central, and posterior regions [4]. The size and connectivity of this tract has encouraged researchers to study it in detail and explore its functions in major brain pathways. One of the findings that makes the CC particularly interesting, in the case of DD, is its involvement in hemispheric lateralization, with studies suggesting the role of callosal organization in the degree of hemispheric lateralization [4].

When it comes to the reading network, research has hinted at an inverse relationship between white matter diffusivity in this tract and reading levels [4,31,32,33], suggesting that less intrahemispheric connectivity helps with better reading skill, partly explained through the working of the left lateralized reading network in typical children. While the CC is not directly associated with the reading network, its suggestive role in hemispherical lateralization makes it an interesting topic of analysis with DD.

#### 1.1.2. White Matter Diffusivity in Typical Readers

In the past couple of decades, there has been a surge in neuroimaging studies that assess white matter diffusivity in typical readers, made possible by the advent of diffusion tensor imaging (DTI). Unlike other neuroimaging methods that regard white matter as a homogenous tissue, DTI has been instrumental in measuring the diffusion properties and directional orientation of white matter tracts [4,34]. For example, extracted from DTI analysis, fractional anisotropy (FA) is one of the most widely used measures of white matter diffusivity, quantifying fiber density through the isotropic movement of water molecules [35].

A vast majority of studies have linked higher FA values in white matter tracts with more efficient language processing and performance in reading-related tasks. A recent MEG and DTI experiment studied the role of ILF in language comprehension and found that higher FA values in the ILF are positively correlated with more efficient semantic processing [36]. Research has found that higher FA values in the SLF correlated with better non-word reading skills in children, suggesting the supporting role of SLF in letter sound correspondence skills that are essential to phonological decoding. Studies also revealed that FA in the UF correlated with reading comprehension in poor readers who were inferred to rely more on individual tract resources and better performance on working memory scores [29,30]. A meta-analysis by Vandermosten et al. [4] reported consistent findings regarding higher FA in CC being inversely related with reading and/or phonological skills [31,33].

### 1.2. Neurobehavioral Abnormalities in the Reading Network in Children with DD

Neuroimaging studies that compare brain activation in children with typically developed reading skills are in contrast to those in children with DD that have shown reduced activation in the typical left lateralized reading network during reading tasks [4]. Neuroscience research over the past two decades has shown some important results: children with DD and other reading disabilities show under-activation in the left tempo-parietal and ventral occipito-temporal regions, providing evidence for the disruption, or lack of development, of the left lateralized reading network, both dorsal and ventral, in children with DD [16,37,38]. Interestingly, studies have also shown that children with DD can sometimes have hyperactivation in their homologous right hemisphere network, including the frontal and prefrontal dorsal sites, which has been suggested as forming the basis of a compensatory reading pathway [4,16,38]. There remains little doubt that both children and adults with DD show different patterns of activation in the brain when compared to typical readers. The reduced activation in key language and reading processing areas of the brain has led researchers to hypothesize that children with DD might not only have reduced activation in such brain areas but also lack efficient connectivity by the underlying white matter tracts between these areas.

A longitudinal study investigated white matter diffusivity in children developing DD as well as children developing typical reading skills and found lower FA in the SLF IV/AF of children who developed DD prior to the onset of reading instruction, the difference being consistent throughout reading development [39]. Research has not only found reduced FA in the left SLF segments but also abnormal orientation of the right SLF segments in children with DD [40]. Research by Steinbrink et al. [41] showed that decreased FA in SLF IV/AF is negatively correlated with the reading skills of children with DD.

While research points towards a clear role of the dorsal white matter reading tract, SLF IV/AF, in reading and DD, studies are more inconsistent when it comes to ventral tracts such as the ILF. However, some studies show abnormalities, including decreased FA, in the left ILF of dyslexic individuals when compared to controls, hinting at the tract’s importance in efficient reading [42,43,44]. Similar results demonstrated a reduced leftward asymmetry of ILF in children with DD [44]. Research has also found that reading comprehension correlated with white matter diffusivity in the UF in poor readers and males with DD, suggesting delayed development of white matter in dysfluent readers [30,44,45]. Studies like these provide additional evidence for the abnormalities in the typical left lateralized ventral reading network in children with DD.

Despite the CC not being a direct part of the reading network, it works to connect the right and the left hemispheres and has been thus hypothesized to play a role in DD. Researchers have suggested increased callosal connectivity as a result of the typical disruption of the left lateralized reading network in children with DD. Research by Frye et al. [31] demonstrated lower FA in the CC of typical readers compared to individuals with DD. Other studies are also consistent in finding a shape difference in the CC of children with dyslexia, indicating a strong growth factor that coincides with literary acquisition [46,47].

In summary, research is suggestive of white matter diffusivity along the ventral and dorsal white matter reading tracts, being essential for reading and language comprehension. While it remains hard to point out the brain profile of children with DD, neuroimaging research is suggestive of differential white matter diffusivity in the dyslexic brain, especially in the dorsal and ventral reading tracts.

### 1.3. Sex Differences

#### 1.3.1. Sex Differences in Children with Typical Reading Skills

Research on biological sex differences in language processing hints towards a female superiority in performing language tasks along with an advantage in early language development [48]. Cross-cultural research has also indicated a female advantage in executive function, verbal skills, language abilities, and related reading abilities, especially in childhood [49,50,51]. Sex differences in language and reading tasks like these have driven research designed to find a neurological basis for such sex differences in language processing and reading. Differences like these are explained in part because of the bilaterality of language processing in females [52]. One study [48] found that females (age 9–15) had greater bilateral activation in the inferior frontal and superior temporal gyrus, along with greater activation in the left fusiform gyrus, compared to boys during two language tasks that were presented in two modalities. A cross-cultural study examining activation amongst dynamic brain networks during language processing found sex differences in interactions and integration of language regions along with differences in functional segregation, providing a neural basis for differential language processing in males and females [53]. While studies like these suggest the potential differences in the language network employed by females, other research suggests that sex differences in language lateralization may be related to the demands of the distinct language tasks utilized instead of lateralization at the population level [54].

The few studies that have examined sex differences in properties of white matter diffusivity have demonstrated some key biological sex differences [4]. One study revealed that men had higher FA in the left SLF whereas women had higher FA in the CC, reflecting the increased lateralization of the language network in men [55]. Another study showed that white matter density, including part of the SLF IV underlying the superior frontal gyrus, is a significant predictor of speech rates but only in females [56]. Moreover, one study found a negative correlation between verbal comprehension index and FA in left cingulate gyrus supracallosal bundle in males but not in females, and a positive correlation between verbal comprehension index and FA in the callosum forceps in females but not in males [57]. Zhao et al. [58] found a correlation between FA in UF in boys with DD, but not girls.

#### 1.3.2. Sex Differences in Children with DD

When studying the biological sex differences associated with typically developing children’s language and reading abilities, one might correctly expect to find similar behavioral and neurobiological differences in children with reading disabilities like DD. While the literature on sex differences in the behavioral symptoms of DD is limited, it has shown consistent differences between biological sexes. Specifically, males are typically more impaired in their orthographical skills and show lower working memory scores, while females with typical reading skills have an advantage in acquiring reading skills compared to typical males [59,60]. Females with learning disabilities are also better at verbal conceptualization compared to their male peers [60]. Moreover, females display a strength in reading-related processing skills, as males with DD perform lower in working memory and orthographic coding tasks compared to their female counterparts [59]. These findings are supported cross-culturally through studies like that of Giorfre et al. [61], which also found a female advantage in a verbally weighted coding test. Overall, results suggest that females with learning disabilities, including DD and other reading disabilities, have strengths in verbal IQ, working memory, visuospatial skills, verbal conceptualization, and orthographic encoding compared to males with learning disabilities.

Although limited, some studies have attempted to find the neurobiological basis of the differential presentation of DD in males and females. Research by Altarelli et al. [62] found that compared to female controls, females with DD showed a reduced thickness in brain regions in the left hemisphere that are responsive to words. No such difference was found in males. Another study found that compared to controls (matched for sex, age, and handedness), right-handed males with DD have larger posterior regions of the CC [63]. Other studies revealed reduced white matter volume in the brains of females with DD compared to both control females and males with DD, especially in the left hemisphere, along with increased cortical irregularities [10].

### 1.4. Current Study

The literature on biological sex differences and reading development suggests the differential acquisition of reading skills in males versus females, along with the underlying neurobiological variances in the male and female brain. Some of these differences are explained in part as related to a more bilateral reading network in females as well as differential language processing abilities in males and females [52,53]. MRI and DTI analysis have also found key differences in white matter diffusivity, in females versus males, including higher FA in males in left hemisphere language areas, hinting at the increased lateralization of the reading network in this group [4,55]. In addition to biological sex differences in the reading network, research has found abnormalities in the reading network, including lower diffusivity in key white matter tracts in dyslexic individuals [4]. However, studies that explore sex differences in a dyslexic population have been limited, in part because of the overrepresentation of males in most dyslexic samples [64]. The current study aims to explore biological sex differences in white matter diffusivity in typical and DD samples. Males with DD are hypothesized to have reduced white matter diffusivity, as measured by FA and mean diffusivity (MD), in the primary white matter tracts corresponding to the reading network (SLF, ILF, UF), as compared to females with DD and both sexes with typically developed reading skills. Given the role of the CC in hemispherical lateralization and the increased bilaterality of the reading network in females [4,52,53], females with DD are hypothesized to have higher white matter diffusivity, as measured by FA and MD, in the CC, compared to control males, control females, and males with DD.

## 2. Materials and Methods

### 2.1. Participants

The data analyzed in this study are part of a longitudinal reading intervention study that involved 155 children (mean age—9.32) from public and charter school systems in Atlanta, Georgia [30]. The current study includes a subset of subjects (*n* = 87, female = 45) in third and fourth grade with usable structural and diffusion MRI data (Table 1). The study was approved by the Georgia State/Georgia Tech Center for Advanced Brain Imaging Institutional Review Board. Intellectual disability was screened for using the Wechsler Abbreviated Scale of Intelligence—II (full scale, verbal or performance scores ≥80). The study was limited to native English speakers, and children with most other psychological, emotional, or neurological conditions were excluded. Participants were not excluded if they met the study’s criteria for commonly co-occurring conditions like attention-deficit hyperactivity disorder (ADHD) and specific language impairment (SLI). Participants were also screened for any contraindicators such as metallic devices, cardiac pacemakers, or dental implants prior to the MRI scan.

### 2.2. Identifying Dyslexic (DD) and Typically Developing (TD) Readers

As part of the intervention study, participants were asked to complete behavioral/cognitive assessments at baseline before interventions were administered. For this study, these included the standardized reading measures Woodcock Johnson 3rd Edition (WJ-3; Broad and Basic Cluster scores) [66] and the Test of Word Reading Efficiency 2nd Edition (TOWRE-2) [67], and the Standardized Reading Inventory Reading Quotient (SRI-2) [68]. WJ-3 contains two broad and basic clusters, which are used to assess oral language, reading, mathematics, written language, and academic knowledge [69]. TOWRE measures fluency in sight word reading and phonemic decoding skills and is commonly used to diagnose reading disabilities and identify individuals who require intensive instruction in word reading [67].

The behavioral measures used to assess for DD are well-validated measures with good reliability [66,67,70,71,72]. The WJ-3 has been shown to retain median reliabilities higher than 0.8 and shows good test–retest (>0.90) and inter-rater (>0.7) reliability [66,72].

In the current sample, DD was diagnosed if a participant scored below the standard score of 85 on any one of the following reading measures: the broad or basic skills clusters on WJ3 [66], Sight Word Efficiency and Phonemic Decoding Efficiency scores on TOWRE-2 [67], or SRI-2 [68]. Children in the typically developing (TD) group did not meet the criteria for DD or other comorbidities (reading scores > 85, no ADHD, no SLI). Based on their results of the various reading and behavioral assessments, participants were grouped into DD (*n* = 67) and TD (*n* = 20) groups.

Statistical analysis, a MANOVA, was conducted to compare the differences between the scores of both the groups, gender (males and females) and reader status (TD and DD), on WJ-3, TOWRE-2, and SRI-2. Results showed a significant main effect of reader status, with subjects with DD scoring significantly lower than TD subjects on all three assessments including WJ-3 (*F* (1, 83) = 215.44, *p* < 0.01), TOWRE-2 (*F* (1, 83) = 200.02, *p* < 0.01), and SRI-2 (*F* (1, 83) = 103.76, *p* < 0.01). No other significant main or interaction effects were found. These results provide evidence for differences in reading skills between TD and DD subjects.

### 2.3. Scanning and DWI Imaging

Following the reading behavioral/cognitive assessments, participants completed MRI scanning, which included diffusion-weighted imaging (DWI) sequences collected by a 3T Siemens scanner at Georgia State/Georgia Tech Center for Advanced Brain Imaging. Dual DWI sequences were collected using reverse phase encoding. Reverse phase encoding has been found to be useful in acquiring reliable DWI data and correcting the geographical distortion by combining two data sets that are acquired from opposite directions [73].

The following scanning parameters were used: FoV—220 × 220 mm; repetition time TD/TE—8900/97 ms; slices—64; b—1000, 4* b—0; 32 gradient directions; voxel size (isotropic)—2 mm; slice thickness—2 mm. The total DWI acquisition time for the data collection in this study was 10 min. Single-shot echo planar imaging (voxel size = 3.438 × 3.438 × 4 mm; matrix size = 64 × 64; TD = 2000 ms; TE = 30 ms; FoV = 220 mm; flip angle = 80°) was used for acquiring T2* images (32 slices; 4 mm slice thickness; no gap) and anatomical scans (MPRAGE; matrix size = 256 × 256; voxel size = 1 × 1 × 1 mm; FoV = 256 mm; TD = 2530 ms; TE = 2.77 ms; flip angle = 7°) in an axial–oblique orientation parallel to the intercommissural line. After quality assurance of the imaging data, the diffusion MRI data correction and quality assurance software tool TORTOISE version 3 [74] was used to preprocess the DWI data using the T2* structural file and MPRAGE reorientation file. The TORTOISE command DIFF_PREP computed the B-matrix of gradient tables which were then used to correct the motion and eddy current distortion and reorient DWIs into target space.

During the final year of the study, the scanner was upgraded to a PRISMA-Fit (20 channel) from a Trio (12 channel head coil). Inter-scanner differences were accounted for by harmonizing the data using ComBat, a genomic method that was adapted to cross-sectional neuroimaging data [75]. The ComBat harmonization model adjusts for biological covariates by removing the site effects using a linear regression model, in addition to modeling the site-specific and scaling factors and utilizing the Bayes empirical statistical method to improve the site parameter estimations for small sample sizes [76]. ComBat has been shown to reduce the type 1 error rate as compared to unharmonized data when the scanner is included as a covariate [75]. Other data acquisition and scan factors were held consistent throughout the study.

### 2.4. White Matter Tract Reconstruction

FreeSurfer 6.0 was used to process the structural and anatomical data. FreeSurfer is a suite of tools that provides visualization of key brain features including segmentation of white matter fascicles through diffusion-weighted MRI [77,78]. White matter tracts of interest were reconstructed using FreeSurfer’s Tracts Constrained by Underlying Anatomy (TRACULA), a method based on the global probabilistic approach [77]. This software uses a priori information about the relative anatomical structures of pathways and combines it with the FreeSurfer cortical and subcortical segmentations in order to derive tractography of the pathways [77]. The TRACULA pipeline was utilized in this study to construct 15 white matter tracts provided by the software, out of which the FA and MD values were extracted for 5 tracts of key interest that have been shown to be the most relevant to the reading network: the CC, and the bilateral SLF (temporal and parietal bundles), ILF, UF, and CC (forceps major and minor).

### 2.5. Analysis

A series of ANOVA were conducted for comparing DD status (as indicated by group memberships (DD or TD)) and gender status (F/M) for FA and MD values in the left ILF, right ILF, left SLFt, right SLFt, left SLFp, right SLFp, left UF, right UF, CC forceps major, and CC forceps minor. Least Significant Difference (LSD) post hoc analysis was conducted for interpreting interaction effects (*F* (1, 83) = 9.180, *p* = 0.003).

## 3. Results

Results for main effects and interactions for the white matter tracts of interest are presented below.

### 3.1. Inferior Longitudinal Fasciculus (ILF)

**Figure 1:**
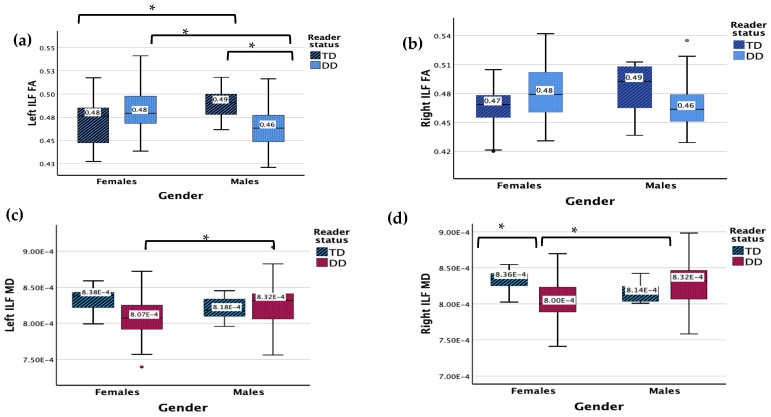
(**a**) Measure of FA for DD and TD groups for the left ILF. (**b**) Measure of FA for DD and TD groups for the right ILF. (**c**) Measure of MD for DD and TD groups for the left ILF. Y-axis numbers are in scientific notation (7.50 × 10^−4^ = 0.00075). (**d**) Measure of MD for DD and TD groups for the left ILF. Y-axis numbers are in scientific notation (7.00 × 10^−4^ = 0.0007). Significant interaction results (*p* < 0.05) are denoted with *. Outliers are denoted with **•**.

There were no significant main effects for DD or gender status for the left ILF FA (Figure 1). The results revealed a significant interaction effect between gender and reader group (*F* (1, 83) = 9.180, *p* = 0.003). Males with DD displayed a lower FA in the left ILF compared to TD males (*p* = 0.042) and females with DD (*p* = 0.003), while TD males showed a higher FA in the left ILF compared with TD females (*p* = 0.042).

There were no significant main effects (RD, gender) for the left ILF MD, but there was a significant interaction between gender and reader group (*F* (1, 83) = 3.955, *p* = 0.050). Males with DD displayed a higher MD compared to females with DD (*p* = 0.005).

The results revealed no main or significant interaction effects for the right ILF FA.

No significant main effects were found for the right ILF MD but there was a significant interaction effect between gender and reader group (*F* (1, 83) = 7.465, *p* = 0.008). Males with DD displayed a higher MD compared to females with DD (*p* < 0.001). Females with DD displayed a lower MD compared to TD females (*p* = 0.017), while no significant difference was found between TD and DD males.

### 3.2. Superior Longitudinal Fasciculus (SLF) Parietal

**Figure 2:**
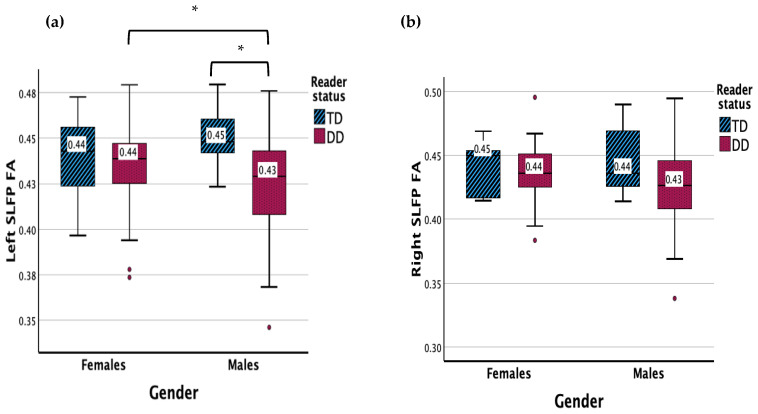
(**a**) Measure of FA for DD and TD groups for the left SLF parietal. (**b**) Measure of FA for DD and TD groups for the right SLF parietal. Significant interaction results (*p* < 0.05) are denoted with *. Outliers are denoted with **•**.

There were no significant main effects for the left SLF parietal FA (Figure 2). There was a significant interaction effect between gender and reader group (*F* (1, 83) = 4.078, *p* = 0.047). Males with DD displayed a lower FA in the left SLF parietal compared to TD males (*p* = 0.005) and females with DD (*p* = 0.047).

Results showed no significant main effects or interaction effect for the left SLF parietal MD.

There were no significant main or interaction effects for the right SLF parietal FA or MD.

### 3.3. Superior Longitudinal Fasciculus (SLF) Temporal

**Figure 3:**
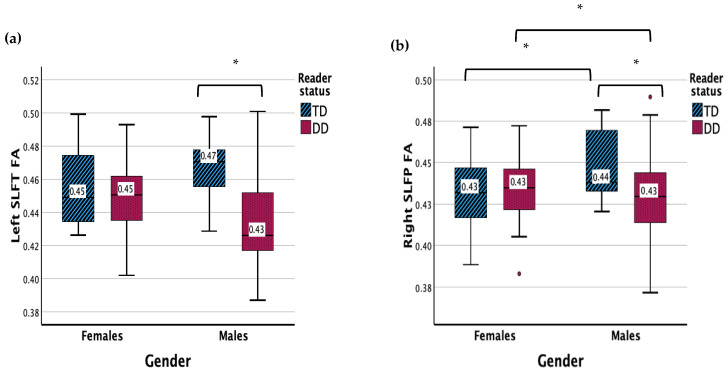
(**a**) Measure of FA for DD and TD groups for the left SLF temporal. (**b**) Measure of FA for DD and TD groups for the right SLF temporal. Significant interaction results (*p* < 0.05) are denoted with *. Outliers are denoted with **•**.

The results revealed a significant main effect of reading status for the left SLF temporal FA (*F* (1, 83) = 6.745, *p* = 0.011) (Figure 3). There was a significant interaction effect between gender and reader group (*F* (1, 83) = 5.416, *p* = 0.022). Males with DD displayed a lower FA in the left SLF temporal compared to TD males (*p* < 0.001) and females with DD (*p* = 0.003).

There were no significant main effects for the right SLF temporal FA. There was a significant interaction between gender and reader group (*F* (1, 83) = 5.539, *p* = 0.021). Males with DD displayed a lower FA in the right SLF temporal compared to TD males (*p* < 0.003) and females with DD (*p* = 0.022), while TD males showed a higher FA in the right SLF temporal compared with TD females (*p* = 0.033).

The results showed no main or interaction effects for the left or right SLF temporal MD.

### 3.4. Uncinate Fasciculus (UF)

There were no significant main or interaction effects for the left UF FA.

There were no significant main effects for the left UF MD but a significant interaction effect for gender and reader group (*F* (1, 83) = 5.105, *p* = 0.026). Females with DD displayed a higher MD in the left UF compared to TD females (*p* = 0.038), while TD females showed a higher UF compared to TD males (*p* = 0.017).

There were no significant main or interaction effects for the right UF FA and MD.

### 3.5. Corpus Callosum (CC)

There was a significant main effect between genders for CC forceps major FA (*F* (1, 83) = 4.091, *p* = 0.046). There were no other main effects or interactions. Females displayed a lower FA compared to males.

There were no significant main effects for CC forceps major MD. There was a significant interaction between gender and reading group (*F* (1, 83) = 6.619, *p* = 0.012). Females with DD showed a lower MD compared to TD females (*p* = 0.033), while no significant difference was found between TD males and males with DD. Females with DD displayed a lower MD compared to males with DD (*p* = 0.037).

Results showed no significant main or interaction effects for CC forceps minor FA and MD.

## 4. Discussion

Reading research has implicated the role of the left lateralized reading network, including its dorsal and ventral components, as being well developed in children with efficient reading and language processing skills, while reading disorders such as DD are often attributed to the disruption of this network [4]. The underlying white matter pathways that connect these critical brain areas form an integral part of this reading network. Neuroimaging research suggests that children with DD have reduced white matter diffusivity in tracts corresponding to the reading network such as the SLF IV/AF [39]. However, it is important to keep in mind that research on reading disorders such as DD remains heavily limited by and possibly biased due to the typically skewed male samples [60]. Studying the brain profile of children with DD along with possible biological sex differences they might present is essential to developing a more complete understanding of the disorder.

The current study hypothesized that males with DD would have reduced white matter diffusivity, as measured by FA and MD, in the primary white matter tracts corresponding to the reading network (SLF, ILF, UF) compared to females with DD and their peers with typically developing reading skills. It is important to highlight that FA and MD are both DTI-obtained but have different interpretations. While FA is a relative measure of diffusion in the principal tract direction, MD is an average of diffusion in all directions [79]. FA results should be interpreted with caution, not falling into the “higher is better” assumption. In reading research, however, subjects with DD have displayed lower FA values compared to non-DD subjects, suggesting that a higher FA in key left hemisphere reading tracts might be indicative of support for more efficient reading network and related skills [4].

The results of the current study generally supported our hypotheses and suggested that males with DD had a lower FA compared to typical males as well as females with DD in the left ILF and left SLF (parietal and temporal regions), whereas no such differences were found in the right ILF or SLF parietal.

Lower FA in the critical white matter tracts corresponding to the left lateralized reading network does suggest less well-developed white matter in these regions in males with DD, whereas the same fails to be true for females with DD in this sample. These results support the hypothesized disruption of the left lateralized reading network for males with DD. Moreover, males with typically developing reading skills had a higher FA compared to females in their left ILF and temporal portions of the right SLF, indicating higher white matter diffusivity in the tracts supporting the left lateralized reading network. As might have also been hypothesized, females with DD did not show a lower FA in their left reading network tracts similar to males. One possible explanation given by previous researchers is that females might at times use an alternative or more bilateral reading network [48,50,51,52].

In contrast to FA, different results were seen for MD. Males with DD displayed a higher MD compared to females with DD in the ILF bilaterally. Additionally, females with DD were found to have a lower MD compared to females with typical reading skills. TD females in the current sample were shown to have a significantly higher MD in the CC compared to DD females. The MD results obtained in this study are congruent with previous research on biological sex differences, finding a higher MD in males compared to females on average in white matter microstructure [80]. However, research on sex differences in DD that uses MD as a measure of white matter diffusivity is very limited, and the few studies that have been conducted fail to show or reproduce a consistent pattern of results [4,80]. A better understanding of the role of MD in DD as well as the role it might play in sex differences require more research.

Given the role of the CC in hemispheric lateralization and the possible increased bilaterality of the reading network in females [4,52,53], females with DD were hypothesized to have higher white matter diffusivity in the CC, as measured by FA and MD. Unexpectedly, in the forceps major region of the CC, females of both groups displayed a lower FA compared to males, and females with DD displayed a lower MD compared to TD females and males with DD. These results possibly suggest a relationship between lower white matter diffusivity in the CC and the potentially increased bilaterality or atypicality of the left lateralized reading network in females. Future research can help further our understanding of the role that CC plays in bilaterality of the left lateralized reading network, along with its implications in DD and sex differences within reading disabilities.

The results of the current study highlight sex differences in pediatric brains that may have important implications in the classroom and other educational settings. Intervention studies have shown that behavioral gains from reading instruction in children are associated with changes in white matter structure and diffusivity, along with increased brain activation in reading-specialized areas [81,82]. DWI studies have linked reading skill acquisition to white matter changes in axonal geometry, myelin remodeling, and diffusivity measured by FA and MD, quantifying white matter plasticity following intervention [83]. Such studies suggest that reading intervention and treatments can help many children with DD, possibly by affecting white matter plasticity.

Given the importance of reading intervention and its possible effect on changes in white matter structure and diffusivity, it is essential to tailor intervention modalities to accommodate for sex differences. Previous research as well as the results of the current study hint at a differential or more bilateral reading network utilized by females. Future research into reading strategies and processes utilized by females with DD can help improve treatment and intervention methods.

Girls have been shown to outperform boys in reading achievement almost globally, making gender inequality in reading acquisition a source of concern for educators [84]. These gender inequalities and differences also show up in reading disorders like DD. Girls with DD perform better at reading tasks compared to boys with DD [59], possibly one of many reasons behind the underdiagnosis of DD in females. These issues outline the importance of studying the underlying neurobiological sex differences in DD, which is a small but essential step towards bridging this gap.

### Limitations

Limitations of this study included its sample of only third and fourth graders (M = 8.82). Although this did not allow for the examination of sex differences in white matter development over time in these children, it is important to note that this age represents a point of critical development, particularly in terms of language and reading [85,86]. It is still important to note that the differences observed in this age group may change over time with continued brain and reading skill development, which might alter sex differences. Additionally, the current sample was relatively small and included children from schools of a particular geographical area, which may limit the generalizability of results. However, the metro area sampled is well documented for its diverse populations [87], and attention was paid during school recruitment to facilitate equal representation of socioeconomic and ethnic groups. Lastly, subjects with DD reported high comorbidity with some other disorders (attention-deficit hyperactivity disorder and specific language impairments), making it difficult to attribute the results to DD alone. However, these comorbidities are representative of the multidimensional nature of developmental disorders in children, with a possible role of white matter in the manifestation of atypical behaviors found across learning and developmental disorders.

The current study utilized DWI imaging and fiber tractography, specifically diffusion tensor imaging. The diffusion tensor model has been criticized because of its oversimplification of the underlying neuroanatomy and high sensitivity to physiological motion, particularly in pediatric samples [88,89], which may affect the accuracy of the measures for white matter diffusivity. However, research has found DWI to be an effective tool in characterizing developing white matter structures in pediatric brains [90,91]. While fiber tractography has been shown to have limitations in differentiating crossing fibers, it is the only technique for visualizing and measuring white matter diffusivity in vivo and non-invasively [4]. The current study utilized FreeSurfer’s TRACULA, which addresses some of these concerns with the use of individualized neuroanatomy. This is particularly advantageous for the measurement of developing white matter in pediatric brain structure.

Additionally, only sex differences were discussed in this article, and this study did not include analysis of other confounding variables. Future research is required to explore the reading network as well as the biomarkers for DD in females.

## 5. Conclusions

Neuroscientific research has relied on DTI measures such as FA and MD to be able to find biomarkers of DD [79]. However, such research has been limited in studying biological sex differences in the DD population in critical white matter tracts, and until recently, a majority of studies relied only on measures of FA, which was assumed to represent white matter integrity [79]. This study provides additional evidence of lower white matter FA in left lateralized reading tracts in males, consistent with the previous suggestions of a possible positive correlation between higher FA in white matter tracts of the left reading network and efficient reading skills. However, this study also highlights the importance of biological sex difference, with females with DD failing to show such differences in the white matter tracts of the left lateralized reading network. Females may use a more bilateral/alternative reading network, which provides one possible explanation of the differences found. In order to study the brain profile of children with DD in its entirety, it is essential to study such sex differences and understand how DD manifests differently in the male and female brain. While this study suggests that females do not show a similar difference in the white matter tracts of the left lateralized reading network to males, further research is required to explore the biomarkers of DD and efficient reading skills in females.

## Figures and Tables

**Table 1:** Subject demographics and test scores.

	DD		TD	
	Males (*n* = 31)	Females (*n* = 36)	Males (*n* = 11)	Females (*n* = 9)
	Mean (SD)	Mean (SD)	Mean (SD)	Mean (SD)
Age	8.97 (0.84)	8.80 (0.71)	8.73 (0.79)	8.78 (0.44)
WASI-2 FSIQ	92.30 (7.35)	91.92 (9.45)	114.10 (11.74)	110.00 (14.49)
WJ3 Basic	82.68 (8.52)	87.72 (7.17)	112.73 (6.54)	110.78 (6.24)
TOWRE-2	69.10 (8.94)	73.31 (8.63)	107.45 (10.45)	103.00 (12.42)
SRI-2	73.78 (12.17)	77.75 (9.82)	105.18 (15.65)	109.33 (15.18)
% With ADHD	51.61%	19.45%	0%	0%
% With SLI	22.58%	27.78%	0%	0%

Subject measures of age in years; Wechsler Abbreviated Scale of Intelligence—II (WASI-2) FSIQ score (scores of general cognitive ability) [65]; Woodcock Johnson 3rd Edition (WJ3) broad and basic reading scores [66]; Test of Word Reading Efficiency 2nd Edition (TOWRE-2) composite score [67]; Standardized Reading Inventory Reading Quotient (SRI-2) composite score [68]; developmental dyslexia (DD); typically developing (TD).

## Data Availability

Data available upon reasonable request and approved data sharing agreement. Data are not publicly available due to ongoing data analysis.
